# Fucoidan inhibits CCL22 production through NF-κB pathway in M2 macrophages: a potential therapeutic strategy for cancer

**DOI:** 10.1038/srep35855

**Published:** 2016-10-24

**Authors:** Jia Sun, Jintang Sun, Bingfeng Song, Lin Zhang, Qianqian Shao, Yanguo Liu, Daoying Yuan, Yun Zhang, Xun Qu

**Affiliations:** 1 Institute of Basic Medical Sciences, Qilu Hospital of Shandong University, Jinan, 250012, Shandong, P. R. China; 2 Laboratory of Neuromuscular Disorders and Department of Neurology, Qilu Hospital of Shandong University, Jinan, 250012, Shandong, P. R. China; 3 Key Laboratory of Precision Biomedicine, Institute of Zhongyuan Biomedical Sciences, Liaocheng People’s hospital, Liaocheng, 252000, Shandong, P. R. China; 4 The Key Laboratory of Cardiovascular Remodeling and Function Research, Qilu Hospital of Shandong University, Jinan, 250012, Shandong, P. R. China

## Abstract

In tumor microenvironment, macrophages as a polarized M2 population promote tumor progression via releasing multiple cytokines and chemokines. A brown seaweed fucose-rich polysaccharide, fucoidan has antitumor activity and immune modulation through affecting tumor cells and lymphocytes. Here, we focused on the effect of fucoidan on macrophages especially M2 subtype. Our results demonstrated that fucoidan down-regulated partial cytokines and chemokines, especially a M2-type chemokine CCL22. Furthermore, fucoidan inhibited tumor cells migration and CD4^+^ T lymphocytes, especially Treg cells, recruitment induced by M2 macrophages conditioned medium through suppression of CCL22. Mechanismly, fucoidan inhibited CCL22 via suppressing p65-NF-κB phosphorylation and nuclear translocation. In addition, p38-MAPK and PI3K-AKT also affected the expression of CCL22 through differential modulation of NF-κB transcriptional activity. Taken together, we reveal an interesting result that fucoidan can inhibit tumor cell migration and lymphocytes recruitment by suppressing CCL22 in M2 macrophages via NF-κB-dependent transcription, which may be a novel and promising mechanism for tumor immunotherapy.

The complex tumor microenvironment plays a critical role in protecting the tumor cells from body repair mechanism and promoting tumor progression, invasion and metastasis, which is comprised of tumor cells, the stroma, blood and lymphatic vessels, and infiltrating immune cells^1^. Infiltrating immune cells largely response to tumor-derived molecular mechanisms, not only exhibit abortive activation, but are co-opted to promote tumor growth^2^. Macrophages are the most common cell population in tumor infiltrating immune cells, which account for 30–50%, known as tumor-associated macrophages (TAMs)^3^.

Macrophages are functionally plastic, and can alter their polarization state to accommodate different physiological conditions. M1 and M2 polarization states are extremes of phenotypic continuum^4^: “classically-activated” macrophages (M1) produce type I pro-inflammatory cytokines, participate in antigen presentation, and have tumoricidal activity. “alternatively-activated” macrophages (M2) produce type II cytokines, promote anti-inflammatory responses, and have pro-tumorigenic functions^5^. Under the influence of T and B cells or cancer cell-derived signals in tumor microenvironment, TAMs mainly exhibit an M2-like phenotype^6^, which promote the formation of blood vessels and lymphatic vessels, enhance tumor cell migration, and tumor proliferation and immune suppression^7^,^8^,^9^. M2 macrophages can be converted into M1, such activation is sufficient on its own to cause tumor rejection^10^. Therefore, the suitable formulation factors targeting immunosuppressive tumor microenvironment and modulating M2 macrophages repolarization or reprogramming may represent a breakthrough for macrophage-directed cancer immunotherapy^11^.

Fucoidan is a fucose-containing sulfated polysaccharide extracted from marine organisms, including brown algae species, which have been marketed as a dietary supplement or nutraceutical^12^. Fucoidan can target multiple receptors, such as scavenger receptors, or signaling molecules in various cell types, including tumor cells and immune cells^13^,^14^. Numerous studies have shown that fucoidan has antitumor effect which is mainly manifested as inhibition of tumor cell growth and migration, promoting lymphocyte proliferation and antitumor cytokine secretion^15^,^16^,^17^. Our previous studies have shown that fucoidan induce a functional maturation of human monocyte-derived dendritic cells^18^ and inhibit monocytes-induced angiogenesis^19^. However, the effect of fucoidan on the properties of macrophages subtypes is poorly understood.

Macrophages are versatile cells characterized by different functional transcriptional profiles in response to microenvironmental signals. Chemokines belong to a superfamily of small proteins with the role of cell chemoattractant in immune and inflammatory reactions, which has been a new dimension of transcriptional profiling to the characterization of different forms of macrophage activation^20^. CCL22 (also called macrophage derived chemokine, a CC-chemokine subfamily member) abundantly released by TAMs^21^, is commonly used as a marker of type M2 macrophages^4^,^22^. A recent study showed that M2 macrophages-derived CCL22 directly promote tumor migration capacities and correlate with venous infiltration^23^. CCL22 selectively recruits CCR4^+^ T lymphocytes (including: Th2 and regulatory T (Treg) cells) to tumor microenvironment through the chemotaxis of CCL22/CCR4 (the receptor of CCL22)^24^. Treg trafficking in tumor microenvironment play a critical role in the maintenance of immunosuppression.

The research adopted the THP-1 (human acute monocyte leukemia cell line) derived macrophages. We found that fucoidan significantly inhibited CCL22 expression in M2 macrophages via NF-κB pathway and further regulated tumor cells migration and lymphocytes recruitment which may represent a new mechanism for fucoidan in antitumor activity.

## Results

### The cytokines transcription of human THP-1-derived macrophages are alerted by fucoidan during polarization process

**Figure 1:**
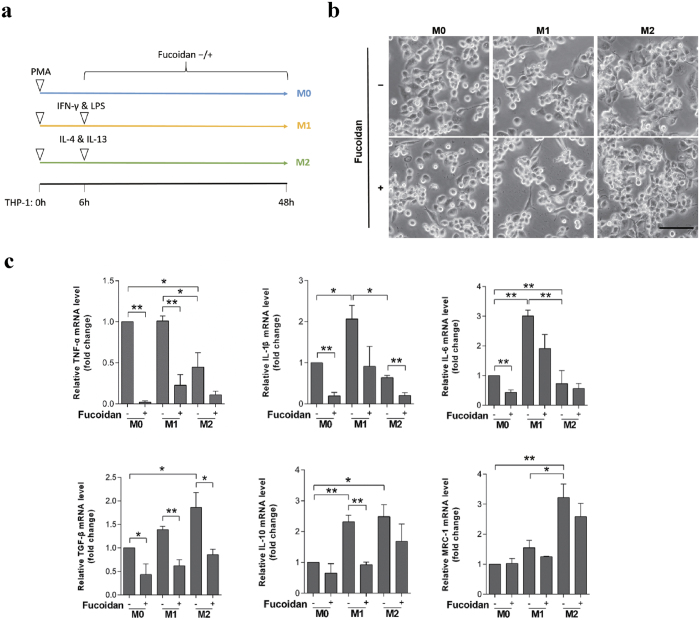
Fucoidan modulates specific gene transcription of human THP-1-derived macrophages during the polarization process. (**a**) A diagrammatic illustration for the macrophage polarization and fucoidan treatment of THP-1 cells. Under PMA (100 ng/ml) stimulation, THP-1 cells differentiated into macrophage-like cells. LPS (100 ng/ml) and IFN-γ (20 ng/ml) stimulated PMA-activated macrophages into M1-phenotype. IL-4 (20 ng/ml) and IL-13 (20 ng/ml) stimulated PMA-activated macrophages into M2-phenotype. (**b**) Morphology of macrophages after 48 h polarization with or without fucoidan treatment. The scale bar represents 50 μm. (**c**) The expression of typical M1/M2 type cytokines and surface marker in non-treated and fucoidan-treated M0/M1/M2-like macrophages were assessed by quantitative real-time PCR and expressed as a fold change compared with M0-like macrophages. M1 biomarkers including TNF-α, IL-1β and IL-6; M2 biomarkers including TGF-β, IL-10 and MRC-1. The values represent means ± SD, n = 3: *P < 0.05, **P < 0.01.

The traditional concepts of M1 and M2 subtypes are seen as two extremes of a continuum of intermediate forms^4^. The effects of fucoidan were detected during macrophages polarization. Fucoidan was added before THP-1-derived macrophages polarization, which is illustrated in the schematic diagram in Fig. 1a. After 48 h polarization, cells were adherent and had a fusiform/fibroblastic appearance. There were no significant differences of morphologic characteristics between fucoidan treated and untreated M0, M1 or M2 macrophage-like populations (denoted as “M0”, “M1” and “M2”, Fig. 1b). mRNA expression of some cytokines and surface marker of M1 and M2 was performed by quantitative real-time PCR. Our results showed that the mRNA expression of TNF-α, IL-1β and IL-6 described as M1 markers tended to be higher in M1 than in M0 and/or M2-like macrophages. The mRNA expression of TGF-β, MRC-1 and IL-10 described as M2 markers were significantly higher in M2 than in M0 and/or M1 macrophages. Fucoidan significantly affected the cytokines transcription and down-regulate TNF-α, IL-1β, IL-6, TGF-β and IL-10 in M0, M1 and/or M2-like macrophages (Fig. 1c).

### Fucoidan regulates the CC chemokines profiles and particularly down-regulate the CCL22 expression and secretion level in M2 macrophages

**Figure 2:**
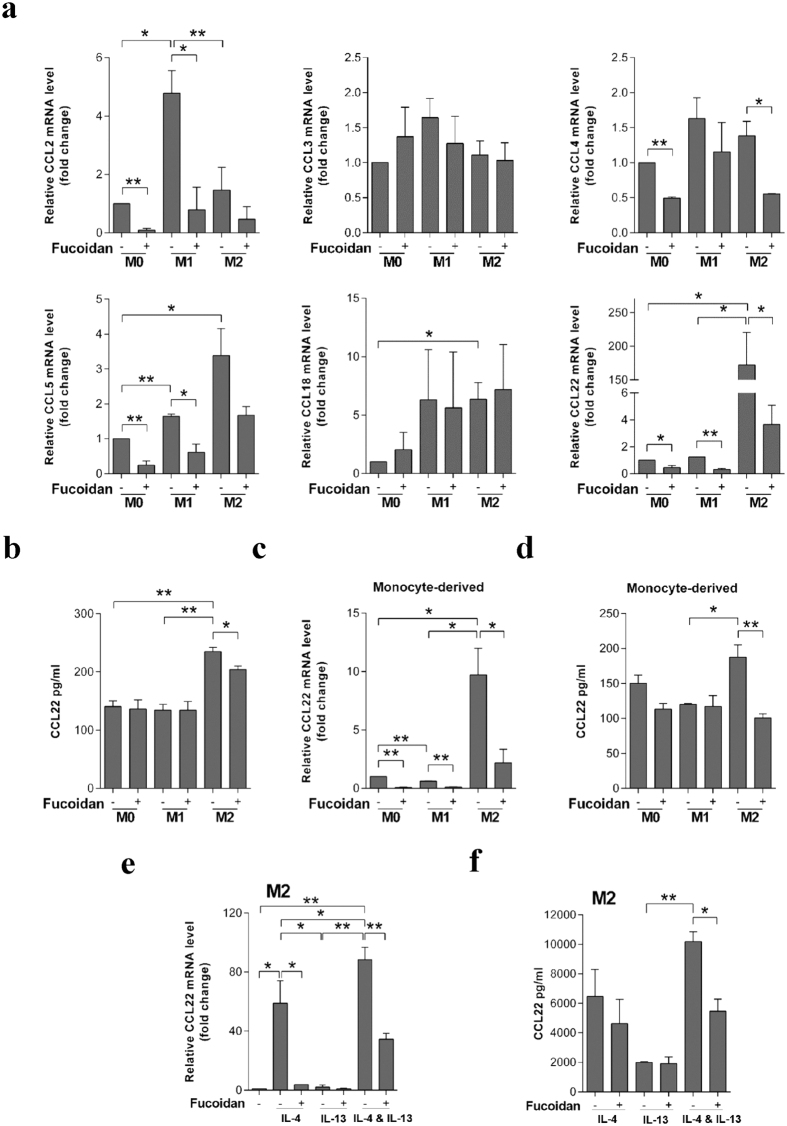
Inhibitory effect of fucoidan on CCL22 production in macrophages. (**a**) Fucoidan modulates CC chemokines transcription of THP-1-derived macrophages during the polarization process. The expression of selected CC chemokines in non-treated and fucoidan-treated M0/M1/M2-like macrophages were assessed by quantitative real-time PCR and expressed as a fold change compared with M0 macrophages. (**b**) Fucoidan modulates CCL22 secretion level of THP-1-derived macrophages during the polarization process. After 48 h polarization, the conditioned media created by non-treated and fucoidan-treated M0/M1/M2-like macrophages were collected. ELISA was then used on the various conditioned media to measure the levels of secreted CCL22. (**c**,**d**) CCL22 transcription (**c**) and concentration in culture media (**d**) of peripheral blood monocyte-derived macrophages. (**e**,**f**) CCL22 transcription (**e**) and concentration (**f**) in non-treated and fucoidan-treated polarized M2 macrophages exposure to IL-4 and/or IL-13. The quantitative real-time PCR data expressed as relative fold change compared with control (polarized M2 macrophages). The value represent means ± SD, n = 3: *P < 0.05, **P < 0.01.

Here we analyzed the mRNA expression of six CC-chemokines including CCL2, CCL3, CCL4, CCL5, CCL18 and CCL22, which primarily are expressed in macrophages. As shown in Fig. 2a, CCL2 and CCL22, which were respectively described as M1 and M2 markers, were significantly higher in M1 or M2-like macrophages. Fucoidan down-regulated the mRNA expression of CCL2, CCL4, CCL5 and CCL22 in M0, M1 and/or M2-like macrophages. It is notable the mRNA level of CCL22 was approximate 50-fold decrease in fucoidan-treated THP-1-derived M2 macrophages (Fig. 2a). The secretion level of CCL22 in the supernatant after 48 h polarization was also decreased by fucoidan in M2-like macrophages (Fig. 2b). The down-regulation of CCL22 by fucoidan was further verified in the primary cells isolated from peripheral blood. After M-CSF treatment, monocytes were differentiated into M0 macrophages *in vitro*. Then stimulated with IL-4 and IL-13, the M0 polarized into M2 subtype. The expression and secretion of CCL22 were rose significantly. Fucoidan notably reduced the expression and extracellular concentrations of CCL22 (Fig. 2c,d). By taking the polarized M2-like macrophages as the research object, the expression of CCL22 was up-regulated by IL-4 and IL-13, significantly. Among them, IL-4 played the major role in the up-regulation of CCL22 expression and secretion. And IL-13 may play a synergic effect (Fig. 2e,f).

### Fucoidan inhibits the effect of M2 supernatant on tumor cell migration and CD4^+^ T lymphocytes recruitment via the down-regulation of CCL22

**Figure 3:**
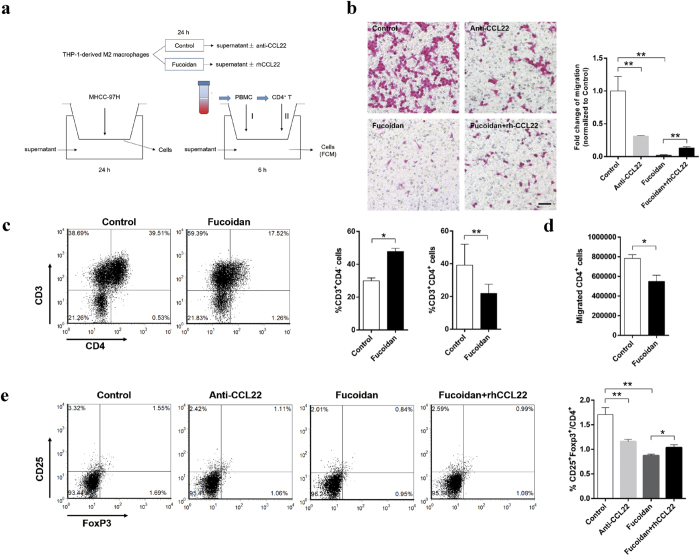
Fucoidan inhibited the migration of tumor cells and affected T lymphocytes recruitment via suppressing CCL22 production. (**a**) Diagram showing the protocol for MHCC-97 migration assay and the PBMC/CD4^+^ lymphocytes recruitment assay. Upper: Supernatants of THP-1-derived M2 macrophages under non- or fucoidan pretreatment were collected and add into lower chamber of transwell with or without neutralizing anti-CCL22 antibody or recombine human CCL22, respectively. Down left: MHCC-97H were seeded in upper chamber. Transwelled cells were stained after 24 h and counted. Down right: PBMC (I) from healthy human peripheral blood separated by lymphocyte separation liquid or microbeads selected CD4^+^ lymphocytes (II) were used as upper chamber components of transwell. Transwelled cells were collected after 6h, and flow cytometry was used to detect the cellular components. FCM: flow cytometry. (**b**) Migration assays were carried out in MHCC-97H cells by using transwell chamber assay. Migrating cells located on the lower surface were fixed in methanol and stained with eosin. The scale bar represents 50 μm. The graph represents fold change. (**c**) Flow cytometry settings used to detect human CD3^+^CD4^−/+^ cells among PBMC recruited to lower chamber contained non-treated and fucoidan-treated THP-1-derived M2 macrophages supernatants from donor human PBMC seeded in the upper chambers (left). %CD3^+^CD4^+^ and %CD3^+^CD4^−^ cells are the percentage of CD3^+^CD4^+^ or CD3^+^CD4^−^ cells among recruited PBMC (right). (**d**,**e**) Flow cytometry settings used to detect human CD25^+^FoxP3^+^ (Treg) cells among CD4^+^ lymphocytes recruited to the bottom chamber filled with conditioned M2 supernatants as tumor cell migration assay. Graphs showed the total numbers of CD4^+^ lymphocytes (**d**) and the flow cytometry (left) and percentage (right) of CD25^+^FoxP3^+^ cells among recruited CD4^+^ lymphocytes (**e**). Control group: the bottom chamber filled with M2 supernatants. The value represent means ± SD, n = 3: *P < 0.05, **P < 0.01 versus the control.

In the above study, we found the strong inhibition of CCL22 by fucoidan in polarizing and polarized M2 macrophages. In order to explore the influence of fucoidan on M2 macrophages functions, the indirect co-culture models based on transwell chambers (Fig. 3a) were established to analyze the tumor cell migration and T lymphocytes recruitment, which were promoted by macrophages-derived CCL22, as described in previous studies.

As shown in Fig. 3b, the migration of MHCC-97H cells (Human hepatoma cells) toward M2 supernatants was significantly reduced when the THP-1-derived M2 macrophages were pretreated with fucoidan for 24 h or CCL22 neutralizing Abs were added to the coculture system. Recombinant human CCL22 (rhCCL22) induced the recovery of MHCC-97H migration ability to some extent in fucoidan pretreated THP-1-derived M2 macrophages supernatants.

Then we identified the population of CD3^+^CD4^−^ and CD3^+^CD4^+^ T cells in PBMC recruited by M2 macrophages supernatants using flow cytometry. The CD3^+^CD4^−^ T cells were more abundant in the cells recruited by fucoidan-preteated-M2 macrophages supernatants. Relatively, the proportion of CD3^+^CD4^+^ T lymphocyte was lower under the chemiotaxis by supernatants from fucoidan stimulated THP-1-derived M2 macrophages (Fig. 3c). To explore whether fucoidan pretreatment of M2 macrophages can inhibit CD4^+^ T lymphocyte migration, we isolated the CD4^+^ T cells from PBMC by immunomagnetic selection using anti-CD4 microbeads for transwell assay. The fucoidan pretreatment of THP-1-derived M2 macrophages reduced the number of CD4^+^ T lymphocytes recruited by M2 conditioned medium (Fig. 3d). As CCL22 is a potent chemokine contributing to Treg cells recruitment, we tried to explore whether fucoidan regulated Treg cells migration mediated by M2 macrophages supernatant. Medium from the fucoidan pretreated THP-1-derived M2 macrophages recruited significantly less CD4^+^CD25^+^FoxP3^+^ (Treg) cells than control medium. The addition of CCL22 neutralizing Abs reduced the preferential recruitment of CD4^+^CD25^+^FoxP3^+^ cells by control medium. And rhCCL22 preferentially recruited CD4^+^CD25^+^FoxP3^+^ cells in fucoidan-pretreated THP-1-derived M2 macrophages supernatant (Fig. 3e), which supporting the idea that CCL22 selectively recruit Tregs.

### CCL22 down-regulation in THP-1-derived M2 macrophages by fucoidan stimulation via NF-κB pathway

**Figure 4:**
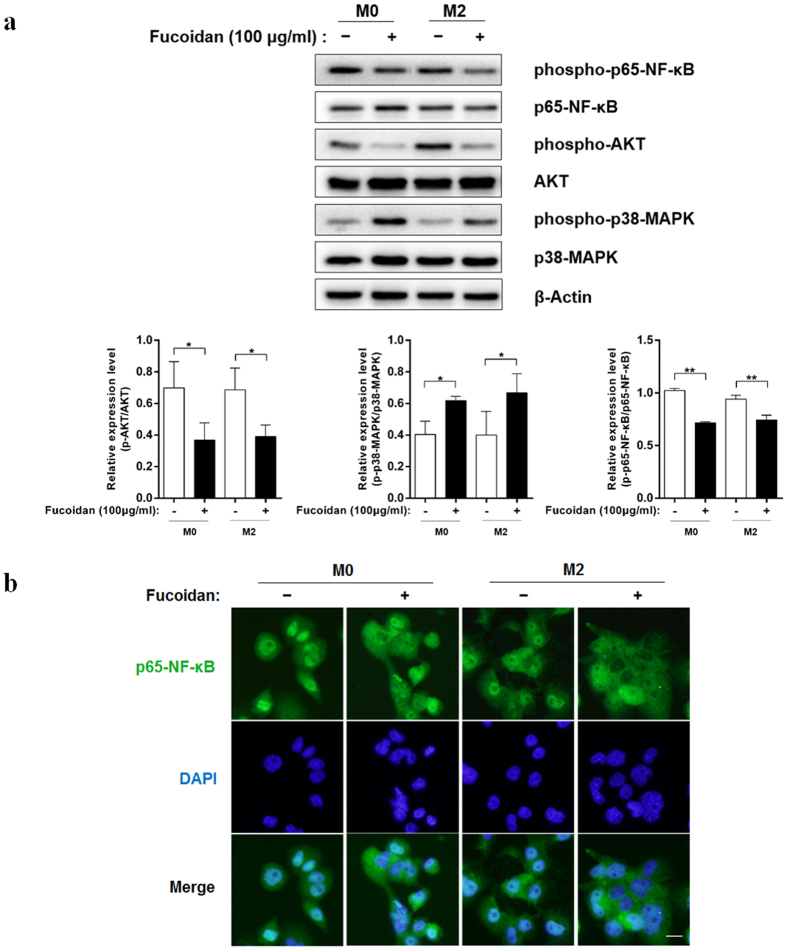
Effect of Fucoidan on the signaling pathways and NF-κB nuclear translocation in THP-1-derived M0 and M2 polarized macrophages. (**a**) M0 and M2 macrophages derived from THP-1 cells were treated with PBS or fucoidan (100 μg/ml) for 1 h. Whole-cell lysates were analyzed by western blot using the appropriate antibodies. The original blot is presented in Supplementary Fig. S1. Densitometry ratios of phospho-p65-NF-κB, AKT and p38 were normalized to the total p65-NF-κB, AKT and p38. The value represent means ± SD, n = 3; *P < 0.05, **P < 0.01 versus the PBS treated group, respectively. (**b**) p65-NF-κB immunofluorescence of THP-1-derived M0 and M2 polarized macrophages, which were treated as described in (**a**). Green (anti-p65-NF-κB) indicates p65-NF-κB distribution, and blue indicates the location of nucleus. The scale bar represents 10 μm.

**Figure 5:**
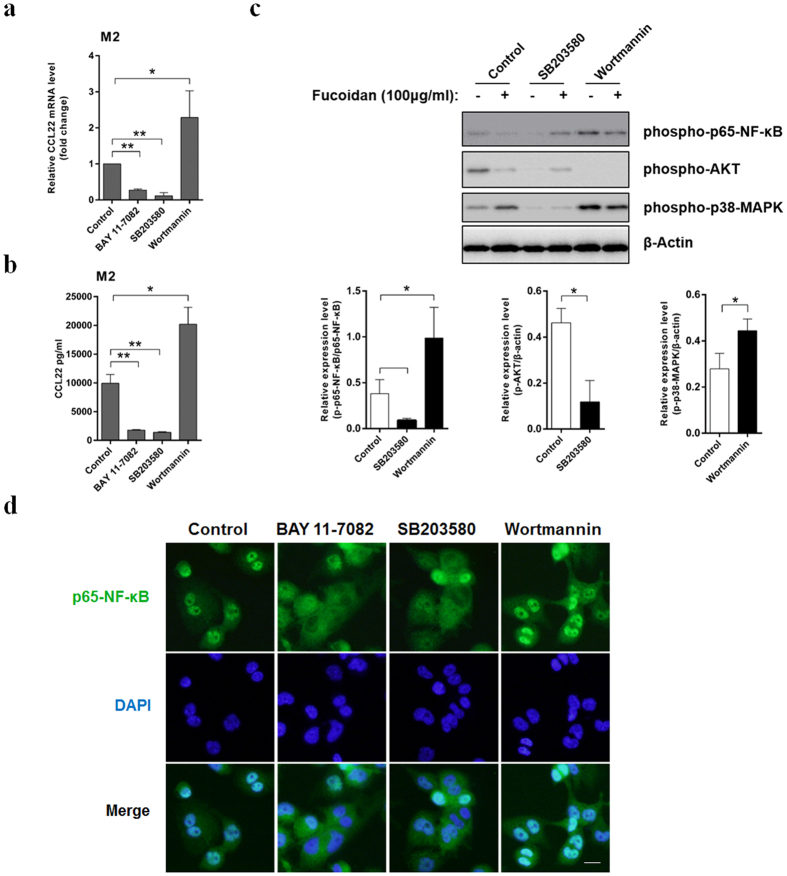
Suppression of CCL22 expression by fucoidan is mediated by a p38- and PI3K-AKT- independent inhibition of the NF-κB pathway. (**a**,**b**) THP-1-derived M2 macrophages were treated with the NF-κB inhibitor BAY11-7082, the PI3K inhibitor Wortmannin or the p38-MAPK inhibitor SB203580 for 24 h. The cells and culture supernatants were collected. CCL22 transcription (**a**) and concentration (**b**) were measured by quantitative real-time PCR and ELISA, respectively. (**c**) M2 macrophages derived from THP-1 cells were pretreated with the Wortmannin or the SB203580 for 30 min, followed by incubation with or without Fucoidan for 1 h. Whole-cell lysates were analyzed by western blot using the appropriate antibodies. The original blots are presented in Supplementary Fig. S2. Densitometry ratios of phospho-p65-NF-κB, AKT and p38-MAPK were normalized to β-actin levels. The cells treated with DMSO were used as control. The value represent means ± SD, n = 3. *P < 0.05, **P < 0.01 versus the control. (**d**) p65-NF-κB immunofluorescence of THP-1-derived M0 and M2 polarized macrophages, which were treated as described in (**c**). Green (anti-p65-NF-κB) indicates p65-NF-κB distribution, and blue indicates the location of nucleus. The scale bar represents 10 μm.

As p38-MAPK, PI3K-AKT and NF-κB signal pathways are affected by fucoidan or engage in expression regulation of CCL22 in other cell types. Phosphorylation of p38-MAPK, AKT and p65-NF-κB were detected to evaluate the mechanism of CCL22 down-regulation by fucoidan in THP-1-derived macrophages. Results from western blot analysis indicated that the phosphorylation of p38-MAPK were up-regulated by fucoidan stimulation in macrophages. At the same time, the phosphorylation of AKT and p65-NF-κB were inhibited (Fig. 4a). Furthermore, we confirmed the inhibition of NF-κB activity by immunofluorescence microscopy assay. As shown in Fig. 4b, treatment of fucoidan induced faint immunostaining of p65-NF-κB translocated into the nucleus. Then we used the inhibitors of PI3K, p38-MAPK and NF-κB to analyze the effect on CCL22 expression and secretion. However, we accidentally found that Wortmannin (PI3K-AKT pathway inhibitor) can significantly increase the expression and secretion of CCL22, p38-MAPK pathway inhibitor SB203580 and NF-κB pathway inhibitor BAY 11-7082 can significantly down-regulate the expression and secretion of CCL22 (Fig. 5a,b). In western blot analysis, Wortmannin which inhibited the PI3K-AKT pathway, promoted the phosphorylation of p65-NF-κB and p38-MAPK. SB203580 which inhibited the p38-MAPK pathway, also inhibited the phosphorylation of p65-NF-κB and AKT at the same time (Fig. 5c). Similar results of NF-κB activation were observed by immunofluorescence (Fig. 5d).

## Discussion

Specific cytokines and chemokines produced by TAMs maintain a suitable microenvironment for tumor growth and progression^21^. Recently, the strategies including blocking macrophages pro-tumor polarization and effector function have all been used successfully in preclinical tumor models^25^. The reprogramming of cytokines and chemokines will result in a progression of functional changes of M1 and M2 macrophages^26^. In this study, we found that fucoidan inhibited partial cytokines and chemokines belonging to either M1 or M2 phenotypes, which suggested that the macrophage repolarization induced by fucoidan was not significant. The down-regulation of cytokines and chemokines probably restrain the effector function of macrophages and maybe even lead to modify the tumor microenvironment. As a chemotactic factor, CCL22 was mainly generated by M2 macrophages^27^, which induced by Th2 cytokines IL-4 and IL-13^28^. We discovered that the transcription of CCL22 was markedly inhibited in fucoidan-treated M2 macrophages during and after polarization, which lead to the low secretion level of CCL22.

CCL22 is highly expressed in a variety of malignant tumors including ovarian tumor, colorectal tumor, breast tumor, hepatocellular carcinoma and follicular lymphoma, and is related to poor prognosis^29^,^30^,^31^,^32^,^33^. It has been recently shown that CCL22 directly induce tumor cells migration^34^. The secretion of CCL22 by macrophages has a chemotaxis effect on Treg cells trafficking, which are suppressive and able to block tumor-specific immunity^35^. We discovered that the tumor cells and Treg cells migration were all inhibited in the conditioned medium from the fucoidan-pretreated M2 macrophages due to the lower concentration of CCL22. These result suggest that the antitumor effect of fucoidan may be potentially via inhibiting CCL22 production by macrophages in tumor microenvironment, which need further *in vivo* investigation.

**Figure 6:**
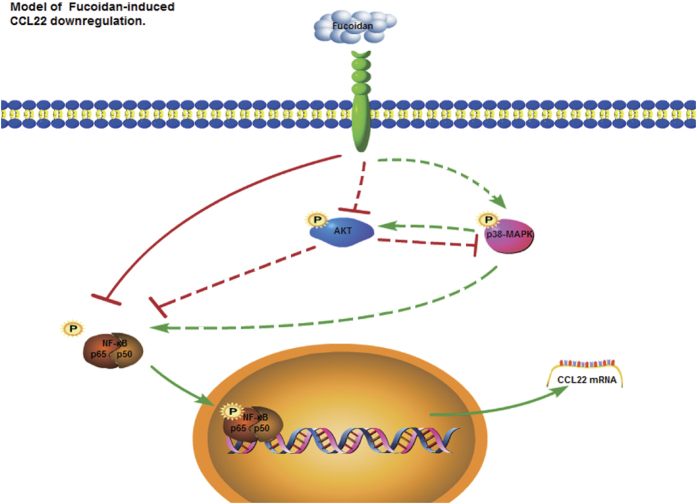
Proposed model for fucoidan-mediated downregulation of CCL22 in macrophages. Green arrow represents activation; truncated red line, inhibition. The dotted lines represent the non-critical effect of AKT and p38-MAPK on fucoidan-mediated downregulation of CCL22.

We next investigated the molecular mechanism of fucoidan-induced CCL22 down-regulation. Previous studies have shown that CCL22 production is dependent on p38-MAPK and NF-κB in various cell types^36^,^37^,^38^. P38-MAPK, PI3K-AKT and NF-κB pathways are influenced by fucoidan^39^,^40^,^41^. In macrophages, p38-MAPK signaling pathway enhances the NF-κB transactivation^42^. PI3K-AKT inactivates the glycogen synthase kinase-β (GSK-β) by inducing its phosphorylation, then inhibit the transactivational activity of NF-κB^43^. Surprisingly, we found that fucoidan significantly up-regulated the phosphorylation of p38-MAPK, inhibited the phosphorylation of AKT and the activity of p65-NF-κB in THP-1-derived macrophages. Further experiments using the signal pathway inhibitors showed the contrary effects of p38-MAPK and PI3K-AKT pathway on NF-κB activation, which induced or inhibited the expression and secretion of CCL22. Analysis of the promoter region of CCL22 showed that the NF-κB binding site in the CCL22 promoter region is essential for the induction of CCL22 expression^37^. Therefore, although fucoidan activated p38-MAPK and inhibited the PI3K-AKT pathway, the production of CCL22 was finally inhibited via the suppression of NF-κB activity (Fig. 6). NF-κB plays an essential role in modulating antitumor immunity^44^. NF-κB activation accounts for increased transcription of several hundred genes. A reduction of myeloid NF-κB activity is associated with tumor surveillance and a reduction in tumor growth, which is likely due to decreased expression of several genes, whose products enhance tumor development, rather than a single one^45^. So we speculate the antitumor activity of fucoidan targeting macrophages may depend primarily on the suppression of NF-κB activity.

Based on the chemotherapy-induced immune suppression, the combination of immunotherapy, which mainly depend on the action of T cells and natural killer (NK) cells, and chemotherapy would be not advantageous for improving therapeutic effect. Macrophages might be more resistant to chemotherapy than other immune cells, and TAMs even promote tumor chemoresistance^46^. Immunotherapy target the activation of macrophages might therefore be synergistic with chemotherapy^47^. Thus, as a marine natural drug, fucoidan could be used as a promising antitumor agent targeting macrophages for tumor immunotherapies.

## Materials and Methods

### Abs and reagents

Recombinant human CCL22, M-CSF, IFN-γ, IL-4 and IL-13 were purchased from R&D Systems (Minneapolis, MN, USA). Fucoidan (from *Fucus vesiculosus*), PMA, LPS, Wortmannin, SB203580 and BAY 11-7082 were purchased from Sigma-Aldrich (St Louis, MO, USA). Monoclonal antibody to CCL22 and the isotype control were purchased from R&D Systems.

### Cell culture

The human monocytes cell line THP-1 was purchased from American Type Culture Collection (ATCC, Rockville, MD, USA). Human hepatoma cell line MHCC-97H was purchased from Liver Cancer Institute of Fudan University (Shanghai, China). Cells were grown in RPMI 1640 medium (HyClone, Logan, UT, USA) containing 10% fetal bovine serum (FBS) (Gibco, Grand Island, NY, USA), 100 U/ml penicillin and 100 μg/ml streptomycin (Invitrogen, Carlsbad, CA, USA) at 37 °C and 5% CO_2_ in a humidified incubator.

### Differentiation and polarization of THP-1 and monocyte-derived macrophages

THP-1 cells were differentiated into M0 by PMA (100 ng/ml) treatment for 48 h in RPMI 1640 medium supplemented with 10% FBS. For M1 subtype polarization experiments, after 6 h PMA treatment, 100 ng/ml LPS and 20 ng/ml IFN-γ were add for another 42 h. For M2 subtype polarization experiments, after 6 h PMA treatment, 20 ng/ml IL-4 and IL-13 were add for another 42 h^48^. Monocytes from PBMC were differentiated into unpolarized macrophages by M-CSF (50 ng/ml) stimulation for 6 days in RPMI 1640 medium with 10% FBS, and then polarized to M1 with LPS and IFN-γ or M2 with IL-4 and IL-13 for another 2 days.

### RNA extraction, reverse transcriptase and quantitative Real-time polymerase chain reaction

Total RNA was extracted by Trizol reagent (Sigma-Aldrich) and reverse transcribed to cDNA, as described previously^49^. Quantitative real-time PCR was performed using SYBR Green real-time PCR Master Mix (Toyobo, Osaka, Japan) with 1 μg cDNA as the template and the primers (shown in Supplementary Table S1) on a LightCycler^®^ 480 qPCR machine (Roche Diagnostics, Penzberg, Germany). Gene expression data were analyzed with LightCycler^®^ software vision 4.0.

### Enzyme-linked immunosorbent assay

The CCL22 concentration in the culture supernatant was measured by ELISA kit (Catalog No: DMD00, R&D Systems) and followed the protocol by the manufacturer’s introduction.

### Preparation and isolation of PBMC and cell subtypes

Human peripheral blood mononuclear cells (PBMCs) were isolated from leukocyte-enriched buffy coats of healthy donors by Ficoll (Sigma-Aldrich) density gradient centrifugation. The collection and use of blood complied with relevant guidelines and institutional practices from the Ethics Committees of Qilu Hospital of Shandong University. Written informed consent was obtained from all subjects. Our study was specially approved by Ethics Committees of Qilu Hospital of Shandong University (Ethical approval No. KYLL-2013-069). For monocytes isolation, monocytes were isolated by magnetic separation using the Miltenyi Monocyte Isolation kit (Miltenyi Biotec, Bergisch-Gladbach, Germany) according to the manufacturer’s instructions, and cultured in 6-well plates at a density of 2 × 10^6 ^cells/well in RPMI 1640 medium supplemented with 10% FBS. CD4^+^ T lymphocytes were purified from PBMC by CD4 Microbeads (Miltenyi Biotec), as described by the manufacturer. More than 98% of the isolated cells were positive for CD3 and CD4, as measured by flow cytometry using APC- or FITC-conjugated Abs against CD3 (OKT3) and CD4 (OKT4) (Biolegend, San Diego, CA, USA).

### *In vitro* migration assay

Supernatants from THP-1 derived M2 macrophages with or without fucoidan pretreatment (24 h), recombination human CCL22 (100 ng/ml) or blocking mAbs (1 μg/ml) were plated into the bottom of migration chambers of 24-well plate. For tumor cell migration assay, 5 × 10^4^ MHCC-97H cells in 200 μl RPMI 1640 medium containing 0.1% FBS were added to the top of 8 μm-pore Costar Transwell chambers (Corning Life Sciences, Corning, NY, USA). After incubated at 37 °C for 24 h in an atmosphere containing 5% CO_2_, the cells on the top side of filter were wiped off with cotton swab, and migrating cells located on the lower surface were fixed in methanol and stained with eosin. For lymphocyte migration assay, 4 × 10^6^ fresh human PBMCs or 2 × 10^6^ isolated CD4^+^ T cells in 200 μl RPMI 1640 medium containing 0.1% FBS were seeded in the top of the 5 μm-pore Costar Transwell chambers (Corning Life Sciences). Following 6 h incubation at 37 °C, cells migrating to the lower chambers were collected and used for flow cytometry.

### Flow cytometry

Migrated cells were collected and stained with a mixed solution of FITC-conjugated mAbs against CD4, PerCP-conjugated mAbs against CD25 (M-A251) and APC-conjugated mAbs against CD3 (Biolegend) for 20 min. Then cells were pretreated with FoxP3 staining buffer set (Catalog No: 00-5523-00, eBioscience, San Diego, CA, USA) according to the manufacturer’s instructions and stained with PE-conjugated mAbs against Foxp3 (206D, Biolegend). Cells were analyzed with a FACS Calibur flow cytometer (Becton-Dickinson (BD), San Jose, CA, USA).

### Western blot analysis

Cells in six-well plates were washed with PBS and lysed with equal volumes of RIPA buffer containing 1 mM PMSF on ice and then centrifuged for 10 min at 12000 rpm under 4 °C. Lysates with equal amounts of protein were separated by 10% SDS-PAGE and then transferred onto PVDF membrane (Millipore, Bedford, MA, USA). The membranes were blocked for 1 h at room temperature with 5% BSA in TBS containing 0.1% Tween20 and then incubated overnight at 4 °C with the following primary antibodies: phospho-Ser536-NF-κB p65, NF-κB p65, phospho-p38-MAPK, MAPK, phospho-AKT, AKT (Cell Signaling Technology, Danvers, Mass, USA); β-actin (Santa Cruz Biotechnology, Santa Cruz, CA, USA). The membranes were exposed to horseradish peroxidase-labeled secondary antibodies (1:3000) for 1 h at room temperature and detected by enhanced chemiluminescence detection system (Amersham Imager 600, GE Healthcare Life Sciences, Little Chalfont, UK and ChemiDoc™ Touch Imaging System, Bio-Rad, Hercules, CA).

### Immunofluorescence staining

For immunofluorescence analysis of nuclear translocation of NF-κB p65, macrophages were fixed in 4% paraformaldehyde for 30 min and incubated with anti-p65 NF-κB antibody (1:400, Catalog No: 8242, Cell Signaling Technology) overnight at 4 °C. The next day, cells were washed three times in PBS, and then incubated with anti-rabbit Alexa Fluor 488-conjugated secondary antibody for 1 h at room temperature. Nuclear was stained by DAPI for 5 min at room temperature. The location of NF-κB was detected by immunofluorescence microscopy (Olympus IX81).

### Statistics analysis

The software SPSS 19.0 was used for statistical analysis. All data were presented as means ± SD. Two-tailed Student’s *t*-test or one-way ANOVA analysis was used to determine significance. P < 0.05 was considered statistically significant.

## Additional Information

**How to cite this article**: Sun, J. *et al*. Fucoidan inhibits CCL22 production through NF-κB pathway in M2 macrophages: a potential therapeutic strategy for cancer. *Sci. Rep.* **6**, 35855; doi: 10.1038/srep35855 (2016).

## Supplementary Material

Supplementary Information
