# Supplementary material for: Fucoidan inhibits CCL22 production through NF-κB pathway in M2 macrophages: a potential therapeutic strategy for cancer
Source: Sci Rep. 2016 Oct 24;6:35855. doi: 10.1038/srep35855 (PMC5075786; doi:10.1038/srep35855)
Supplement: Supplementary Information [file srep35855-s1.pdf]

Supplementary Information

Fucoidan inhibits CCL22 production through NF-κB pathway in M2 macrophages: a potential therapeutic strategy for cancer

Jia Sun[1,4], Jintang Sun[1, *], Bingfeng Song[1], Lin Zhang[1], Qianqian Shao[1], Yanguo Liu[1], Daoying Yuan[3], Yun Zhang[2], Xun Qu[1,2,3, *]

[1]Institute of Basic Medical Sciences, Qilu Hospital of Shandong University, Jinan, 250012, Shandong, P.R. China

[2]The Key Laboratory of Cardiovascular Remodeling and Function Research, Qilu Hospital of Shandong University, Jinan, 250012, Shandong, P.R. China

[3]Key Laboratory of Precision Biomedicine, Institute of Zhongyuan Biomedical Sciences, Liaocheng People's hospital, Liaocheng, 252000, Shandong, P.R. China.

[4]Laboratory of Neuromuscular Disorders and Department of Neurology, Qilu Hospital of Shandong University, Jinan, 250012, Shandong, P.R. China

[*]Correspondence and requests for materials should be addressed to X.Q. (email: quxun@sdu.edu.cn) and J.T.S. (email: sunjintang003@163.com)

Supplementary Table S1 List of primers used in Quantitative RT-PCR

**Primer name**		**Sequence**
TNF-α	Forward	5'-CAG AGG GAA GAG TTC CCC AG-3'
	Reverse	5'-CCT TGG TCT GGT AGG AGA CG-3'
TGF-β	Forward	5'- AAG GAC CTC GGC TGG AAG TG -3'
	Reverse	5'- CCC GGG CCA TGC TGG TTG TA -3'
IL-1β	Forward	5'-AAA CAG ATG AAG TGC TCC TTC CAG G-3'
	Reverse	5'-TGG AGA ACA CCA CTT GTT GCT CCA-3'
IL-6	Forward	5'-CCT TGG GTC CAG TTG CCT TCT-3'
	Reverse	5'-CCA GTG CCT CTT TGC TGC TTT C-3'
IL-10	Forward	5'-GAT CCA GTT TTA CCT GGA GGA G-3'
	Reverse	5'-CCT GAG GGT CTT CAG GTT CTC -3'
MRC-1	Forward	5'-CGA GGA AGA GGT TCG GTT CAC C-3'
	Reverse	5'-GCA ATC CCG GTT CTC ATG GC-3'
CCL2	Forward	5'-CCC CAG TCA CCT GCT GTT AT-3'
	Reverse	5'-TGG AAT CCT GAA CCC ACT TC-3'
CCL3	Forward	5'-GCA ACC AGT TCT CTG CAT CA-3'
	Reverse	5'-TGG CTG CTC GTC TCA AAG TA-3'
CCL4	Forward	5'-GCT TTT CTT ACA CTG CGA GGA-3'
	Reverse	5'-CCA GGA TTC ACT GGG ATC AG-3'
CCL5	Forward	5'-CGT GCC CAC ATC AAG GAG-3'
	Reverse	5'-GGA CAA GAG CAA GCA GAA AC-3'
CCL18	Forward	5'-CTC TGC TGC CTC GTC TAT ACC T-3'
	Reverse	5'-CTT GGT TAG GAG GAT GAC ACC T-3'
CCL22	Forward	5'-CGA GGA AGA GGT TCG GTT CAC C-3'
	Reverse	5'- CAT CTT CAC CCA GGG CAC TCT-3'
GAPDH	Forward	5'-GAA GGT GAA GGT CGG AGT C-3'
	Reverse	5'-GAA GAT GGT GAT GGG ATT TC-3'

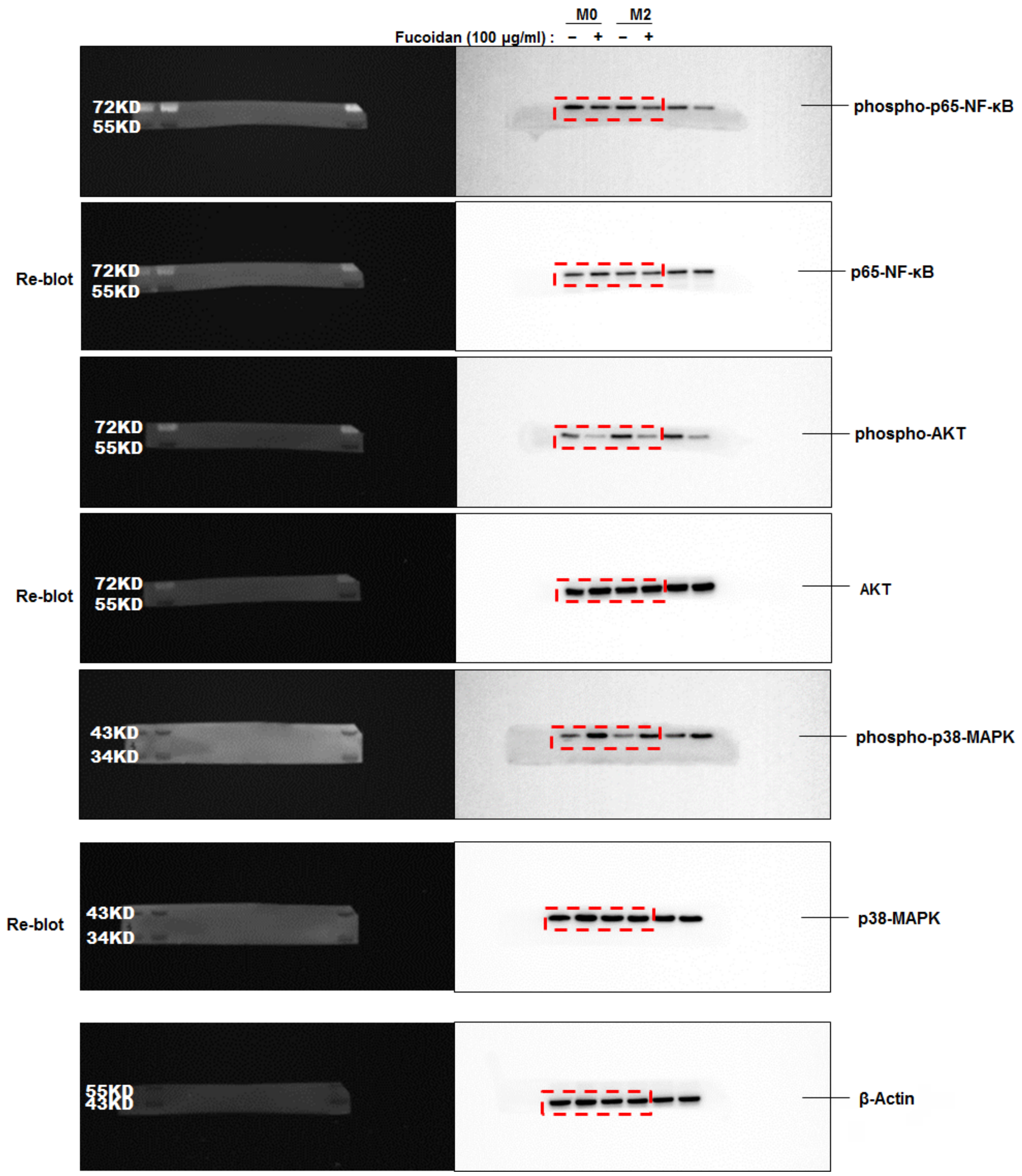

Supplementary Figure S1

The original blot of Figure 4a. M0 and M2 macrophages derived from THP-1 cells were treated with PBS or fucoidan (100 μg/ml) for 1 h. Whole-cell lysates were analyzed by western blot using the appropriate antibodies. The specific protein bands were analyzed using the Amersham Imager 600 (GE Healthcare Life Sciences, Little Chalfont, UK). (Re-blot: The blots were stripped in re-blot plus strong solution and probed with either anti-p65-NF-κB, anti-AKT and anti-p38-MAPK.)

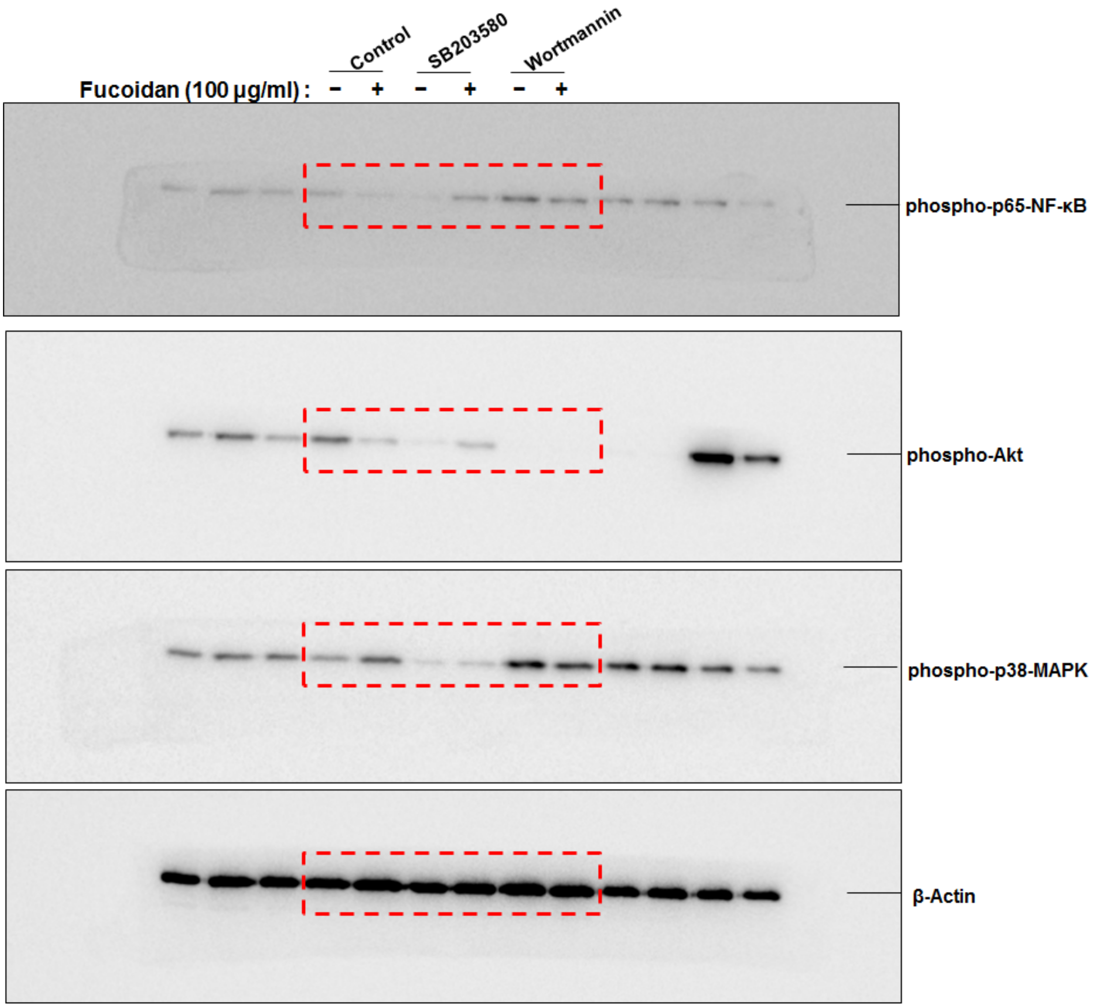

Supplementary Figure S2

The original blot of Figure 5c. M2 macrophages derived from THP-1 cells were pretreated with the Wortmannin or the SB203580 for 30 min, followed by incubation with or without Fucoidan for 1 h. Whole-cell lysates were analyzed by western blot using the appropriate antibodies. The specific protein bands were analyzed using the ChemiDoc™ Touch Imaging System (Bio-Rad, Hercules, CA)
